# Self-Assembly of Model Amphiphilic Peptides in Nonaqueous Solvents: Changing the Driving Force for Aggregation Does Not Change the Fibril Structure

**DOI:** 10.1021/acs.langmuir.0c00876

**Published:** 2020-06-28

**Authors:** Alessandra Del Giudice, Axel Rüter, Nicolae Viorel Pavel, Luciano Galantini, Ulf Olsson

**Affiliations:** † Department of Chemistry, Sapienza University of Rome, P. le A. Moro 5, Rome 00185, Italy; ‡ Division of Physical Chemistry, Lund University, Lund SE-22100, Sweden

## Abstract

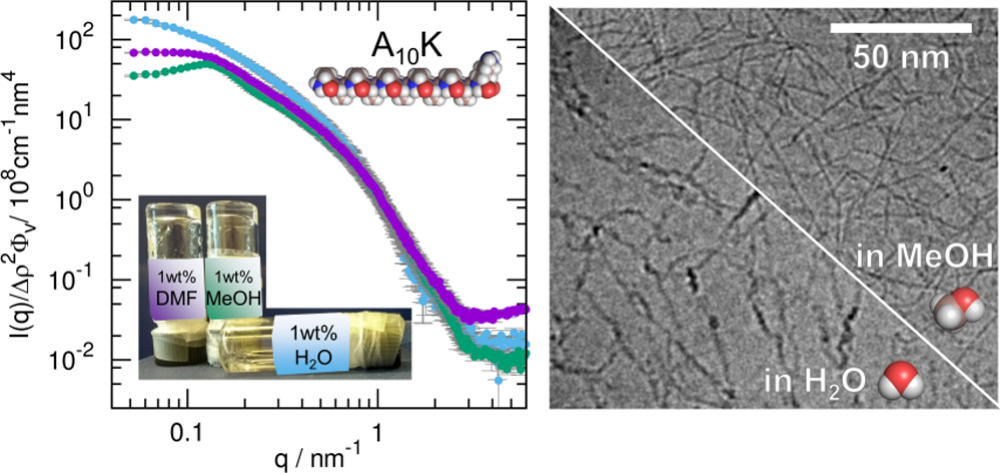

Within the homologous series of amphiphilic peptides A_*n*_K, both A_8_K and A_10_K self-assemble in water to form twisted ribbon fibrils with lengths around 100 nm. The structure of the fibrils can be described in terms of twisted β-sheets extending in the direction of the fibrils, laminated to give a constant cross section of 4 nm by 8 nm. The finite width of the twisted ribbons can be reasonably explained within a simple thermodynamic model, considering a free energy penalty for the stretching of hydrogen bonds along the twisted β-sheets and an interfacial free energy gain for the lamination of the hydrophobic β-sheets. In this study, we characterize the self-assembly behavior of these peptides in nonaqueous solutions as a route to probe the role of hydrophobic interaction in fibril stabilization. Both peptides, in methanol and *N*,*N*-dimethylformamide, were found to form fibrillar aggregates with the same β-sheet structure as in water but with slightly smaller cross-sectional sizes. However, the gel-like texture, the slow relaxation in dynamic light scattering experiments, and a correlation peak in the small-angle X-ray scattering pattern highlighted enhanced interfibril interactions in the nonaqueous solvents in the same concentration range. This could be ascribed to a higher effective volume of the aggregates because of enhanced fibril growth and length, as suggested by light scattering and cryogenic transmission electron microscopy analyses. These effects can be discussed considering how the solvent properties affect the different energetic contributions (hydrophobic, electrostatic, and hydrogen bonding) to fibril formation. In the analyzed case, the decreased hydrogen bonding propensity of the nonaqueous solvents makes the hydrogen bond formation along the fibril a key driving force for peptide assembly, whereas it represents a nonrelevant contribution in water.

## Introduction

Understanding the driving forces for peptide self-assembly is of both fundamental and practical significance. These aggregation processes are relevant in the formation of protein- and peptide-based amyloid fibrils involved in both diseases and functional aspects of biological systems.^1,2^ Peptide self-assembly also represents a versatile tool for building structural elements made of designed peptide building blocks for advanced biomaterial applications.^3−5^ In addition, for a growing field such as peptide-based therapeutics, knowing the phase behavior and stability of various peptide solutions and formulations is of outmost importance.^6^ Because of the multifaceted chemical nature of the natural amino acids composing the peptide building blocks, their self-assembly is dictated by various noncovalent interactions (hydrophobic effect, hydrogen bonding, electrostatic interactions, and π–π stacking) and their interplay.^7^ Their relative importance will depend on the peptide sequence as well as the properties of the surroundings, determining a complex free energy landscape with deep valleys corresponding to low free energy states.

Short amphiphilic peptides, in which a “tail” of apolar amino acids is flanked by a “head” of charged residues, can be thought as structurally related to conventional surfactants and have been the object of systematic studies aimed at revealing structure–property relationships.^8,9^ In water, the main driving force for self-assembly is the hydrophobic effect driving the nonpolar amino acids to aggregate in order to minimize the hydrophobic–water interaction. Like conventional surfactants, many amphiphilic peptides have displayed well-defined critical aggregation concentrations below which the monomeric solution state is favored. However, for some other experimental observations, the picture of an equilibrium micellization is not appropriate. Instead, the self-assembly has been suggested to occur through crystallization with the processes of nucleation and growth of ordered aggregates.^10^ Indeed, the most remarkable difference between the self-assembly of conventional surfactants and short amphiphilic peptides is the participation of abundant hydrogen bonding interactions in the latter, in particular between the amide groups of peptide backbones. This typically leads to the formation of β-sheets extending along the direction of hydrogen bonding, which further order into one-dimensional nanostructures.^11^

**Figure 1:**
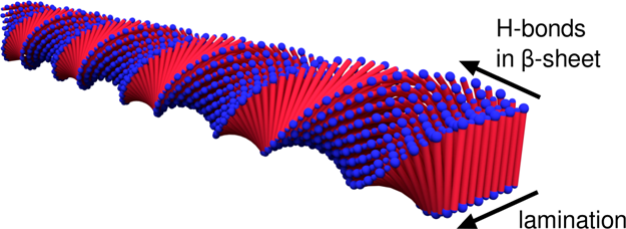
Schematic image showing the geometry of an A_*n*_K peptide twisted ribbon consisting of nine laminated β-sheets. The peptide molecule is represented as a red stick with two blue polar heads of different size. The directions in which the β-sheets extend in length and in which they laterally laminate are highlighted with arrows.

The A_*n*_K model peptides belong to this class of amphiphilic peptides, with a sequence of *n* hydrophobic alanine residues (A) and a single polar lysine residue (K) at the C terminus bearing a positively charged amino group, in addition to the unprotected N-terminus. Within the homologous series with *n* = 6, 8, and 10, the heptapeptide A_6_K was shown to form hollow nanotubes above a critical aggregation concentration,^12^ whereas the longer homologs A_8_K and A_10_K self-assembled at progressively lower critical concentrations into twisted ribbons with a well-defined cross section.^13^ Despite the different overall morphology, the systems showed a similar local packing of the peptides assembled in laminated antiparallel β-sheets that generate a two-dimensional oblique crystal lattice.^14^ In particular, the aggregation of A_8_K and A_10_K into fibrils can be described as β-sheets extending in the direction of the fibril length axis, which laminate laterally by coming in contact with their methyl group-rich surfaces, because of the hydrophobic effect (Figure 1). The natural twisting of the chiral building blocks, which was observed experimentally in the ribbons, provides an explanation for the finite cross section of the aggregates.^15,16^ A simple model considering a free energy penalty due to stretching of hydrogen bonds within the β-sheets arising from the twist of the ribbons and an interfacial free energy gain of the hydrophobic β-sheets, which would favor their lamination, was able to reasonably predict the optimal width of the twisted ribbons.^17^

Characterizing the behavior of these peptides in a less polar solvent could be a route to probe the interfacial energy contribution of hydrophobic side chains exposed to the solvent considered in this model. Following an analogy between the amphiphilic peptides and the conventional surfactants, one could hypothesize that the solvophobic effect in nonaqueous solvents should be weaker, resulting in an increase of the critical aggregation concentration^18−20^ and a decrease of the equilibrium size of the aggregates.^21^ In our investigation, we considered the self-assembly of the peptides in two nonaqueous solvents: methanol (MeOH), which can be considered the first more hydrophobic analogue of water in which one hydrogen atom is replaced by a methyl group, and *N*,*N*-dimethylformamide (DMF), a solvent commonly used in peptide synthesis and considered to be “aprotic” because it is only a hydrogen bond acceptor.

Even if MeOH in some contexts can be considered a nonaggregating solvent,^22^ this is not the case for peptide self-assembly. For a peptide containing a diphenylalanine moiety, a different molecular packing was observed, leading to a morphological transition from fibrils (water) to nanotubes (MeOH).^23,24^ For “designer” peptides, which aggregate into β-sheet twisted fibrils, MeOH was observed to enhance β-sheet formation even at low concentrations, at which the peptide was soluble in water as a random coil.^25^ There is some interest in understanding peptide self-assembly also in organic solvents. This can tell us about the likely effect of a more hydrophobic environment on peptide self-assembly, with possible implications for the issue of lipid environments in amyloid fibril formation.^26,27^ Such knowledge can also expand the range of applications these peptidic building blocks can be explored for.^28,29^

## Methods

### Sample Preparation

The synthetic peptides A_*n*_K (*n* = 8 and 10) were acquired from CPC Scientific Inc. as trifluoroacetate (TFA)-stabilized salts with purities >96% and were used without further purification. Ultrapure water (Milli-Q; Millipore), MeOH (purity 99.8%, VWR chemicals), and DMF (purity 99.5%, Fischer or RPE, ACS for analysis, Carlo Erba Reagents) were used as solvents. Weighted amounts of lyophilized peptides powders were solubilized with weighted amounts of solvents, and the sample concentration was expressed in wt %, determined excluding the mass of the counterion. Unless otherwise specified, initial solutions were prepared at a concentration around 1 wt %, and after 8 h from the initial solubilization, they were stepwise diluted to obtain the samples at lower concentration. For converting mass fractions to volume fractions (Table S1, Supporting Information), the values of the peptide densities in water previously reported^13^ were assumed (1.50 g cm^–3^ for A_8_K and 1.26 g cm^–3^ for A_10_K) and the solvent densities at 25 °C were used (*d*_water_ = 0.997 g cm^–3^, *d*_MeOH_ = 0.786 g cm^–3^, and *d*_DMF_ = 0.948 g cm^–3^^30^).

### Small-Angle X-Ray Scattering Experiments and Data Analysis

Small-angle X-ray scattering (SAXS) and wide-angle X-ray scattering (WAXS) measurements were performed on a SAXSlab Ganesha pinhole instrument, JJ X-ray System Aps, equipped with an X-ray microsource (Xenocs) and a two-dimensional 300k Pilatus detector (Dectris Ltd., Switzerland). The X-ray wavelength was λ = 1.54 Å. Samples prepared at 1 wt % were measured in quartz capillary cells at room temperature in an evacuated chamber. Images were collected at three given sample-to-detector distances, and the azimuthally averaged intensities as a function of the scattering vector *q* = (4π/λ) sin(2θ), where 2θ is the scattering angle, were subtracted for the contribution of the capillaries filled with solvent and put to absolute scale by calibration against water. Additional experiments on dilution series were performed at the SWING beamline of Synchrotron SOLEIL (Gif-sur-Yvette, France), with an X-ray wavelength λ = 1.033 Å. The sample-to-detector distance was 308 cm, allowing data collection in the scattering vector range 0.023 < *q* < 3.9 nm^–1^. The measurements were performed by loading the samples in disposable quartz capillaries with 2.0 mm diameter; scattering frames were collected with 10 exposures of 490 ms on an Eiger 4M detector (Dectris). The data collected on capillaries filled with solvent were used for subtraction. The SAXS data reduction (radial integration, absolute scaling, frames averaging, and background subtraction) was performed using the FoxTrot software developed at the SOLEIL synchrotron. The subtracted scattering profiles on absolute scale were analyzed by both applying model-independent approaches (Guinier fit using Primus,^31^ indirect Fourier transform using BayesApp^32^) and fitting with the form factor of a long elliptical cylinder^33^ with homogeneous electron density.^34^

### Circular Dichroism Measurements

Circular dichroism (CD) experiments were performed using a JASCO J-715 CD spectrometer. The spectra in the range 185–260 nm were collected at room temperature with 1 nm band width, 2 s response time, and 20 nm min^–1^ scan rate. The average of three scans was used for each spectrum. Samples of A_8_K and A_10_K at a concentration of 0.5 wt % in water and MeOH were placed in a 0.01 mm path length quartz cuvette (Hellma) for measurement. The solvent baseline was subtracted. Data in millidegrees were converted to units of mean residue ellipticity (deg dmol^–1^ cm^2^) considering a peptide concentration of 0.005 g cm^–3^, the path length, the molecular weights (715 g mol^–1^ for A_8_ and 857 g mol^–1^ for A_10_K), and the number of residues (9 for A_8_ and 11 for A_10_K).

### Cryogenic Electron Microscopy Imaging Experiments

Cryogenic electron microscopy imaging (Cryo-EM) was performed on a JEOL JEM-2200 instrument using a TVIPS F416 camera at the national Center for High Resolution Electron Microscopy in Lund University. An accelerator voltage of 200 kV was used. The samples were vitrified on lacey carbon film-covered copper grids using a Leica EM GP automatic plunge freezer. Grids used in all EM measurements were glow-discharged for increased wettability before sample preparation.

### Light Scattering Measurements

Dynamic light scattering (DLS) experiments were performed with an ALV/DLS/SLS-5022F goniometer system with an ALV-7004 correlator at a scattering angle of 90°. The laser source was a 22 mW He–Ne laser with a wavelength of 632.8 nm. The samples were placed in 5 mm glass tubes, kept at 25 °C, and the intensity autocorrelation functions were collected over runs of 300 s, using two avalanche photodiode detectors operating in the pseudo-cross-correlational mode. Light scattering experiments were also performed using a modulated three-dimensional light scattering instrument (LS Instruments GmbH, Switzerland), implementing a laser with a wavelength of 660 nm and two avalanche photodiodes. For the estimate of solubility, the mean count rate, at 90°, was obtained as the average of three subsequent runs of 100 s. The experimental intensity correlation functions *g*^(2)^(τ) were converted to *C*(τ) = (*g*^(2)^(τ) – 1)/β = |*g*^(1)^(τ)|^2^,^35^ where β is an instrumental constant, close to unity, and *g*^(1)^(τ) is the correlation function of the electric field, which was fitted using the cumulant expansion to obtain an average diffusion coefficient.^36^ Fitting was performed with an in-house MATLAB script.

## Results and Discussion

**Figure 2:**
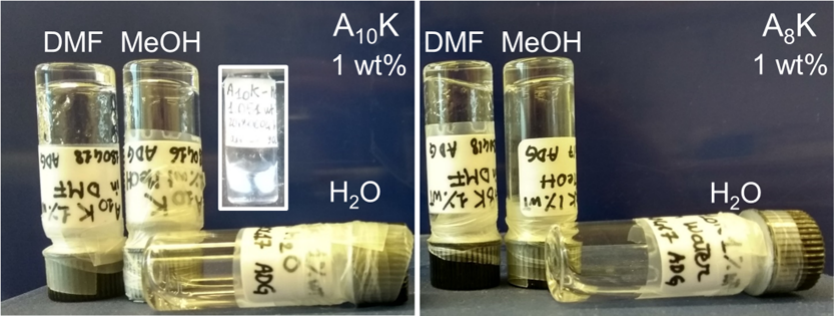
Arrested dynamic state of A_10_K (left) and A_8_K (right) peptides in MeOH and DMF: photograph of 1 wt % peptide samples in vials after 1 week from direct preparation. In the inset, photograph of the A_10_K 1 wt % sample in MeOH between cross-polarizers.

To study the self-assembly of A_8_K and A_10_K in nonaqueous solvents and compare it to the behavior in water, an initial concentration of 1 wt % was chosen. Previous SAXS experiments on the peptides in water^13,17^ have shown that this concentration could be considered dilute enough not to manifest any effects of interaggregate interactions in the scattering profiles. The first observation made was that the peptide samples in MeOH and DMF were more viscous than the corresponding samples in water prepared at similar concentrations and presented highly viscous (gel-like) states after 5–12 h from solubilization (Figure 2). Corresponding states have been observed for A_10_K samples in water only above a volume fraction of 0.02 (around 3 wt %).^37^ Among the prepared samples, the 1 wt % A_10_K solution in MeOH also exhibited birefringence (Figure 2).

**Figure 3:**
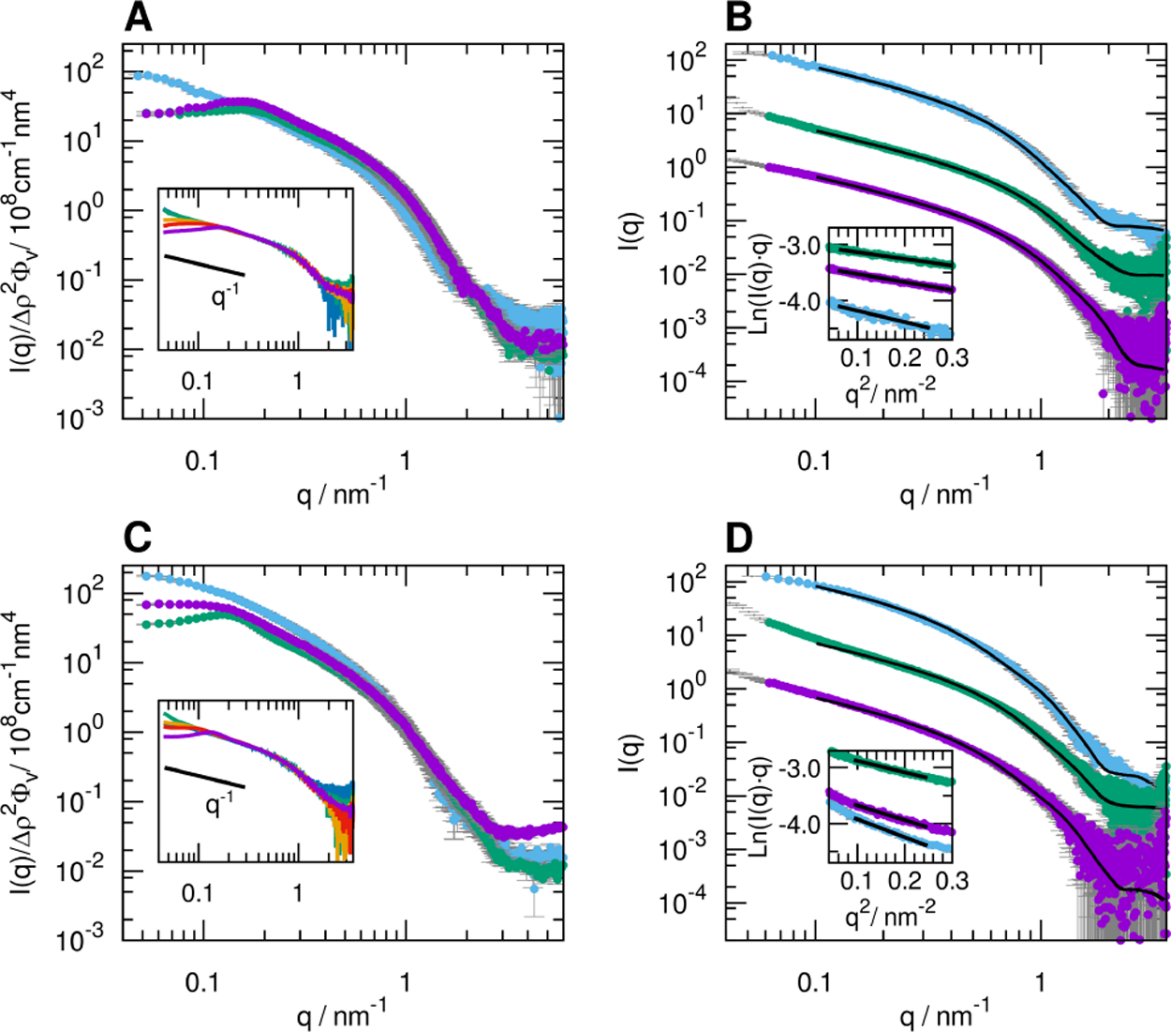
SAXS profiles of A_8_K (A) and A_10_K (C) in water (blue), MeOH (green), and DMF (purple). The peptide concentration is around 1 wt %, and the scattered intensity in cm^–1^ is divided by the estimated peptide volume fraction and by the squared scattering length density difference Δρ^2^, in nm^–4^. In the insets, the SAXS profiles of A_8_K and A_10_K solutions in MeOH at concentrations of 1 wt % (purple), 0.5 wt % (red), 0.25 wt % (orange), 0.125 wt % (green), and 0.0625 wt % (blue) are reported. The data were multiplied by a scaling constant chosen to ensure superimposition in the *q* range 0.635–1.227 nm^–1^ (listed in Table S1, Supporting Information, together with concentration values). The SAXS experimental data at a peptide concentration of 1 wt % in water (blue) and 0.125 wt % in MeOH (green) and DMF (purple) are reported for A_8_K and A_10_K in (B,D), respectively, along with the calculated curve based on the form factor of a cylinder with an elliptical cross section (black lines) and the Guinier fits for rodlike objects in the insets. The curves are shifted by a convenient factor to avoid overlapping. The optimized parameters of the calculated curves are shown in Table S2, Supporting Information.

SAXS experiments (Figure 3) showed very similar scattering profiles in the three solvents, indicating that aggregates formed in MeOH and DMF essentially have the same ribbon morphology as in water.^13,17^ A correlation peak in the lower *q* range was observed for the samples in MeOH and DMF, whereas this feature was absent in the water samples (Figure 3A,C). This observation testifies the presence of characteristic distances in the fibril suspension arising from interaggregate interactions, suggesting that the samples in MeOH and DMF, at these concentrations (volume fraction around 0.006), are not in the dilute regime, but rather in the semidilute regime, borrowing terminology from the theoretical framework for polyelectrolytes.^38,39^ Synchrotron SAXS experiments on dilution series for A_8_K and A_10_K in MeOH confirmed that the correlation peak disappears at low enough concentration (insets in Figures 3A,C and S1, Supporting Information). Therefore, the SAXS data collected for samples in MeOH and DMF at a concentration below 0.25 wt %, in the low *q* regime, clearly present the *q*^–1^ slope characteristic of rigid rodlike objects and could be interpreted in terms of a particle form factor similarly to what has been suggested^17^ for 1 wt % samples of A_8_K and A_10_K in water (Figure 3B,D and Table S2, Supporting Information).

The SAXS data could therefore be described by means of the form factor of an elliptical cylinder^33^ whose length was set to a value beyond the maximum distance accessible with the available minimum *q*, except for the A_10_K fibrils in water, which had already shown to have a shorter length, around 60 nm, when prepared at this concentration (Table S2, Supporting Information).^13,17^ The optimized values for the cross-sectional semiaxes agree with an approximate diameter of 6 nm, as previously reported.^13,17,37^

However, when inspecting the parameters obtained by fitting several experimental data, we consistently detected slightly smaller cross-sectional sizes for the aggregates of the same peptide in the nonaqueous solvents compared to water. This observation of a smaller average cross section was confirmed by estimating the radius of gyration of the cross section, *R*_CS_, by applying a Guinier fit for rodlike objects (insets in Figure 3B,D and Table S3, Supporting Information). A second estimate of the same parameter could be derived from the pair distance distribution function of the cross section (Figure S2, Supporting Information) obtained by indirect Fourier transform of the *I*(*q*)*q* versus *q* curve at *q* > 0.3 nm^–1^.

Some geometrical considerations can be made based on the assumption that the fibril cross section is given by the lamination of a constant number of extended peptide molecules. In the model used for fitting the SAXS data, the cross section of the aggregates is approximated as a homogeneous ellipse of semiaxes *a* and *b*. The smaller axis, 2*a*, would represent the cross-sectional thickness and considered equal to the length of the fully extended peptide, estimated as 3.2 nm for A_8_K and 3.9 nm for A_10_K. The cross-sectional width, 2*b*, should be given by the number of laminated β-sheets times the separation between them, estimated to be 0.54 nm.^14^ Considering how the radius of gyration depends on the semiaxes *a* and *b* of a homogeneous elliptical cross section *R*_CS_ = (*a*^2^ + *b*^2^)^(1/2)^/2,^40^ we tried to estimate the fibril width (Table S3, Supporting Information). These estimates would suggest that the number of laminated β-sheets in the fibrils decreases to approximately 80 and 90% of the value in water in MeOH and DMF, respectively. The difference between the average fibril cross-sectional radius of gyration in water and MeOH, although small, can be considered statistically relevant; a different slope of the scattering profile in the *q* region 0.3–0.5 nm^–1^ is clearly observable (Figure 3B,D insets).

The simplified model invoked to explain the finite and monodisperse cross section of the A_8_K and A_10_K ribbons^17^ was based on a trade-off between the surface energy gain with lamination of the apolar alanine-rich sheets and the energy penalty due to increased distortion of the hydrogen bonds within the β-sheets. A smaller optimal number of laminated β-sheets in a solvent with lower interfacial tension than water toward a hydrophobic surface (Table 1) would qualitatively agree with this interpretation.

**Table 1:** Some Physicochemical Properties of the Three Solvents in Which the Self-Assembly of the Peptides Was Investigated

property	H_2_O	MeOH	DMF
solubility of NaCl at 25 °C (g/L)^49^	359	14.9	4
solubility of hexane at 25 °C (g/L)^50,51^	0.0124	604	
dielectric constant at 25 °C^52^	78.3	32.6	36.7
dipole moment (Debye)^52^	1.85	1.70	3.82
CMC of SDS at 25 °C (nM)^53,54^	7.8	6.9	14.62
surface tension at 25 °C (dynes/cm)^55^	72.70	22.10	34.40
Hansen solubility parameter (H-bond) δ_H_ (J/cm^3^)^56^	42.3	22.3	11.3

However, the approximation of the peptide ribbons as a homogeneous cylinder with an elliptical cross section neglects both possible electron density variations at the surface of the ribbons and their twisted nature. In particular, the first approximation can be problematic in the comparison between peptide fibrils in water and other solvents because of the complex description of an interface toward the bulk solvent, which includes solvation layers and counterions. It is known that proteins in water have a first hydration shell with electron density ≈10% higher than the bulk solvent.^41^ A similar interfacial effect could occur on the surface of the peptide ribbons in water determining a slight overestimation of the cross section, assuming the simple homogeneous electron density model, compared to the true geometrical size of the assembled peptide aggregates. On the other hand, it could be estimated that undissociated counterions should be present to a larger extent at the surface of the peptide ribbons in the solvents with a lower dielectric constant than water because of stronger Coulombic attraction (Table 1). One can predict that the presence of undissociated counterions should also increase the cross section deduced from the SAXS data compared to the geometrical size of the bare peptide fibrils, whereas the observed values are smaller than those in water. Overall, because of the approximations in the model adopted to describe the SAXS data, we can conclude that the ribbons formed by the self-assembly of A_8_K and A_10_K in MeOH and DMF have a very similar or only slighter smaller cross section compared to the aggregates formed in water.

**Figure 4:**
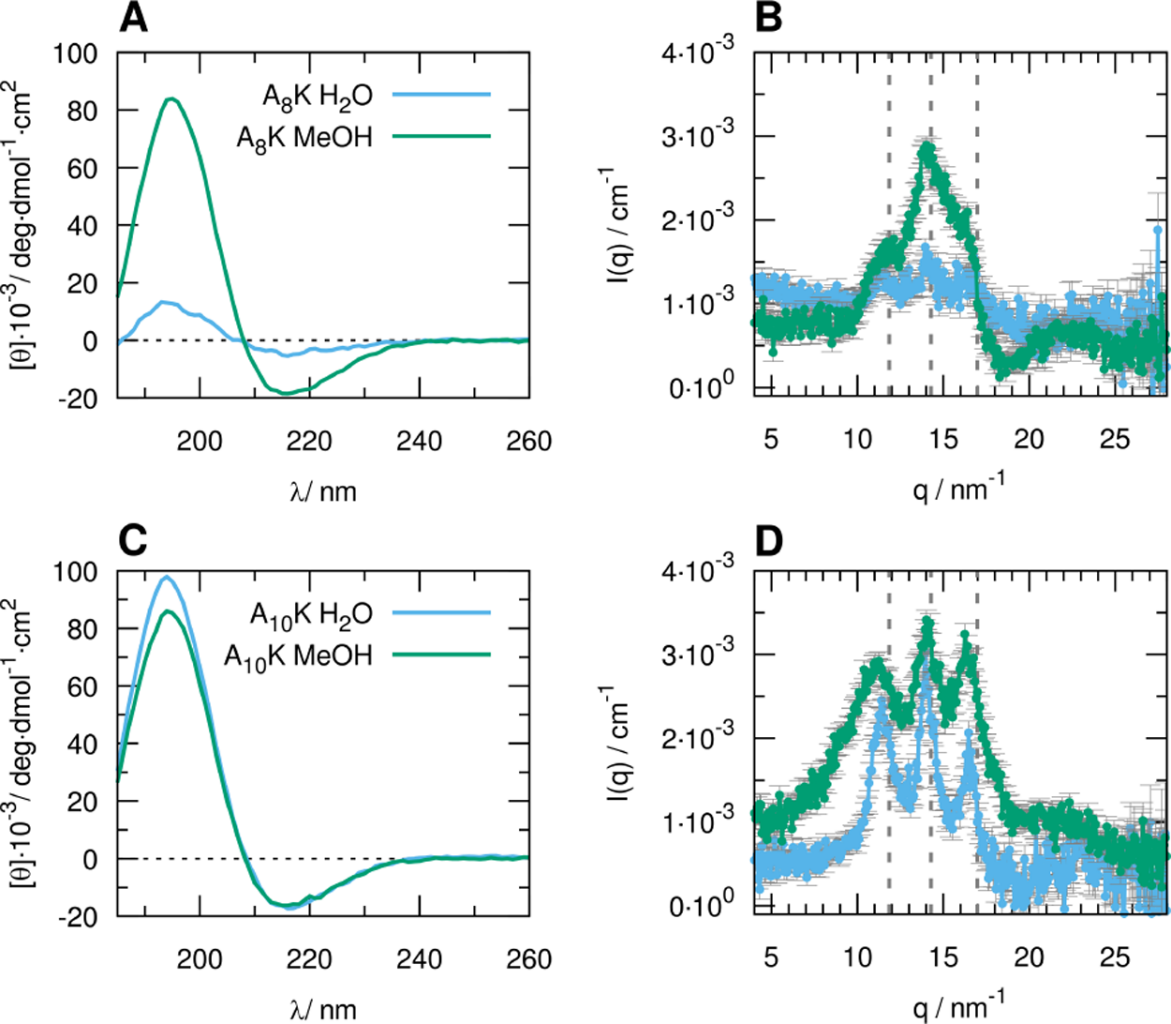
CD spectra of 0.5 wt % A_8_K (A) and A_10_K (C) solutions in water (blue line) and in MeOH (green line). WAXS data of 1 wt % A_8_K (B) and A_10_K (D) solutions in water (blue) and MeOH (green). The positions of reference Bragg peaks reported in the literature^13,14^ are marked with vertical dashed lines in (B,D).

Concerning the local packing of the peptides within the ribbons, previously reported X-ray scattering data in the wide-angle range for A_8_K and A_10_K in water, at concentrations 2–3 wt %, showed three distinguishable peaks at *q* values 11.8, 14.3, and 16.1 nm^–1^.^13^ These three Bragg reflections have been indexed to an oblique two-dimensional lattice,^14^ resembling the molecular packing within polyalanine crystals.^42^ The WAXS data collected on A_10_K in MeOH, at 1 wt %, clearly showed three Bragg peaks at the same position as in water, suggesting the same molecular packing within the fibrils formed in the nonaqueous solvent (Figure 4D) and confirming its crystalline nature. The lower peptide concentration compared to previously published data and the presence of the broad solvent scattering peak having its maximum in the same *q* region where the reflections occur (centered around 13 nm^–1^ for DMF and 16 nm^–1^ for MeOH, whereas it is found around 20 nm^–1^ for water) made the WAXS peaks of the aggregates in the nonaqueous solvents experimentally more difficult to resolve, particularly for the samples in DMF. Some excess signal compared to the solvent in the *q* region 10–18 nm^–1^ could be evidenced for these samples, with the most intense maximum around 14 nm^–1^ (Figure S3, Supporting Information).

For the A_8_K and A_10_K fibrils formed in MeOH, it was also possible to obtain reliable CD data, whereas for the samples in DMF, the solvent absorption in the far UV hampered the use of this spectroscopic technique. The CD spectra (Figure 4A,C) showed the characteristic positive band at λ = 195 nm and a negative band at λ = 215 nm, already observed for the peptide aggregates in water and consistent with a β-sheet peptide arrangement.^43^ It could be noticed that at 0.5 wt % peptide concentration, the CD signal arising from the A_8_K aggregates is much weaker in water than in MeOH. This already suggests a lower solubility of this peptide as a monomer in MeOH compared to water, resulting in a higher aggregate concentration. This is further investigated below.

### Length of Peptide Fibrils

**Figure 5:**
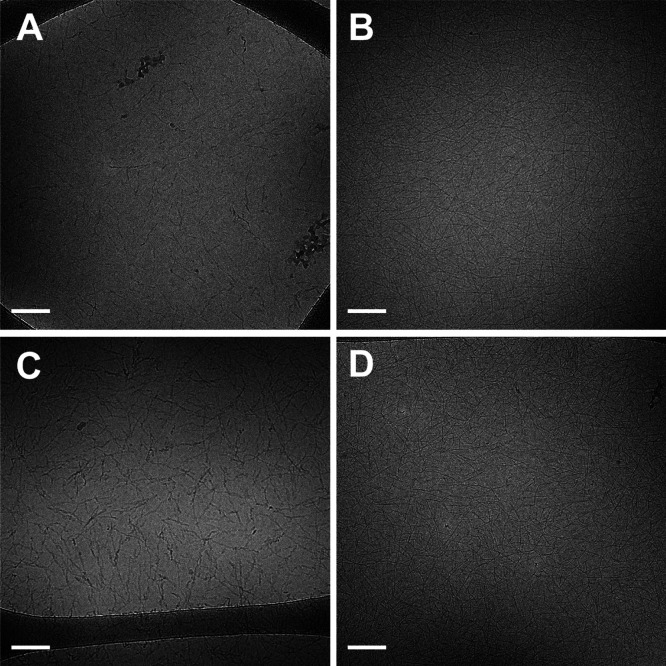
Cryo-TEM images for A_8_K 0.05 wt % in water (A), A_8_K 0.01 wt % in MeOH (B), A_10_K 0.02 wt % in water (C), and A_10_K 0.01 wt % in MeOH (D). All scale bars are 100 nm.

From the data presented in the previous section, we could appreciate that A_8_K and A_10_K form in MeOH fibrillar aggregates with the same local packing as in water, possibly with a slightly smaller cross section. Cryo-TEM experiments confirmed that in water and MeOH, similar fibrils are formed by both A_8_K (Figure 5A,B) and A_10_K (Figure 5C,D). The aggregates have an estimated cross section of approximately 4 nm (Figure S4, Supporting Information), consistent with the SAXS experiments. The images overall suggest that the fibrils of both peptides grow longer in MeOH compared to those in water. This is consistent with the observation that, at the same peptide mass concentration, solutions are more viscous in MeOH than in water (Figure 2).

**Figure 6:**
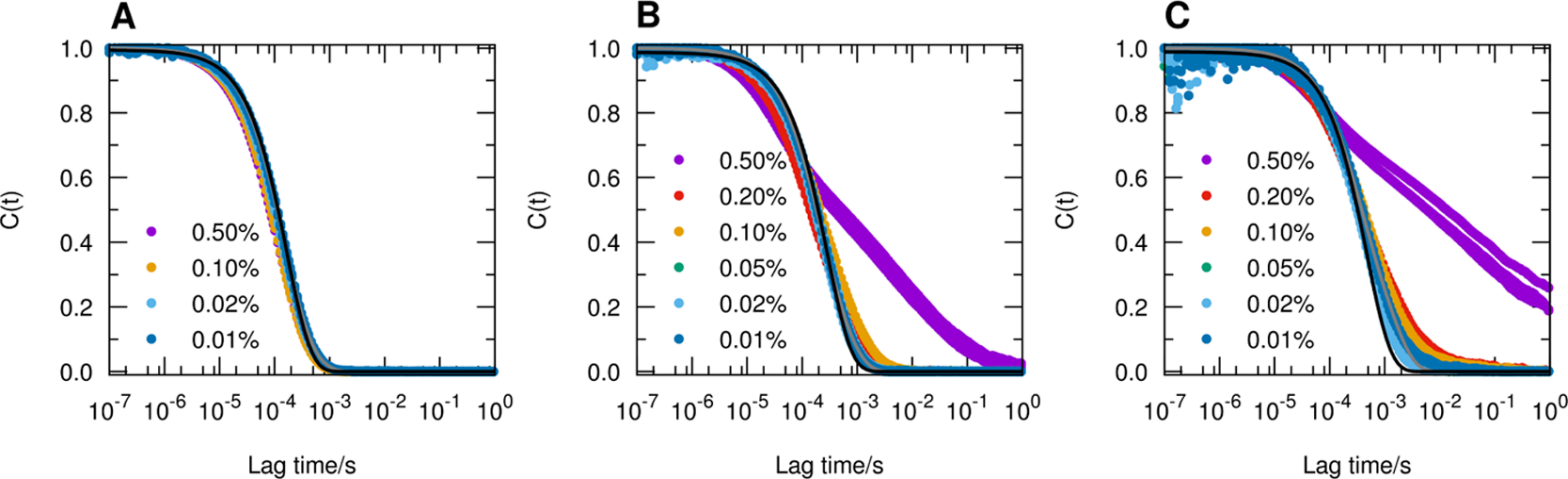
Normalized intensity autocorrelation functions of A_10_K peptide aggregates in water (A), MeOH (B), and DMF (C) as a function of concentration. For the most dilute samples, the data could be described as a double exponential decay (gray line) or using the method of cumulants with average diffusion coefficients of 8.6, 5.0, and 2.7 × 10^–12^ m^2^ s^–1^ in water, MeOH, and DMF, respectively (black lines).

DLS was used to compare the length of the aggregates in the different solvents and to detect the slowing down of the dynamics above the overlap concentration of aggregates. The normalized intensity autocorrelation functions, *C*(*t*), are shown in Figure 6. At low concentrations, the correlation function is approximately unimodal, and an average diffusion coefficient, *D̅*, can be obtained using the method of cumulants.^36^ The relaxation is assumed to be purely diffusive and can be interpreted as a weighted average of the parallel and perpendicular translational modes, giving rise to an average diffusion coefficient *D*_t_ = 1/3*D*_∥_ + 2/3*D*_⊥_.^44^ From the obtained values of the diffusion coefficient, an average length can be estimated by using the equations proposed by Broersma for long cylinders^45−47^
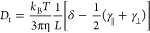
1
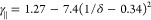
2
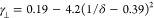
3where *k*_B_ is the Boltzmann constant, *T* is the temperature in kelvin, η is the solvent viscosity, *L* is the rod length, δ = ln(2*L*/*d*), *d* being the cross-sectional diameter, and the γ_∥_ and γ_⊥_ values are expected to be valid for 0.15 < 1/δ < 0.35, that is, approximate aspect ratios 8 < *L*/*d* < 400.^47^ By assuming the viscosity values of η_water_ = 0.891 cP, η_MeOH_ = 0.485 cP, and η_DMF_ = 0.802 cP, and the diameter of the cross section obtained by SAXS data fits, we obtained a Z-averaged length of about 200 nm in water, 800 nm in MeOH, and 1000 nm in DMF. At the highest concentration, 0.50 wt %, in MeOH and DMF, a slow mode is observed in the correlation function. This signals the onset of dynamic arrest and that the system is trapped in a nonergodic glassy state. The same effect is also observed in water but at a concentration ≥1 wt %.^13,37^ Reasonably, this transition occurs at a lower concentration in MeOH and DMF because of the longer aggregates formed in these solvents.

### Monomer Solubility and Driving Force for Self-Assembly

**Figure 7:**
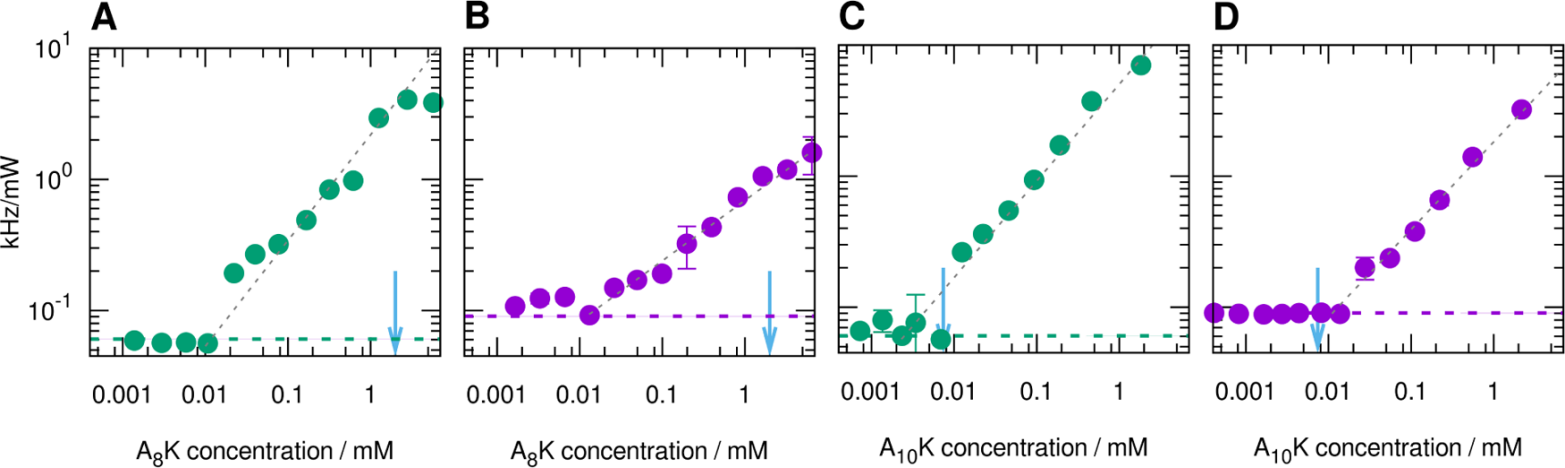
Variation of the scattered light intensity with peptide concentration for A_8_K in MeOH (A) and DMF (B) and for A_10_K in MeOH (C) and DMF (D). The values of the solubility in water estimated with the same approach (from ref (13)) are indicated for comparison with blue arrows. The signal measured for the pure solvent is shown as a horizontal dashed line. The gray dashed lines represent a guide for the eye.

Aggregates begin to form above a particular concentration, which can be interpreted as the monomer solubility, *c*_s_. This concentration can be measured, for example, by following the scattered light intensity as a function of dilution of a sample with an initial concentration *c* > *c*_s_.^13^ For this purpose, solutions of the peptides at 1 wt % were gradually diluted in order to determine at which concentration the light scattering signal reached the solvent level, which is a good indication of the monomer solubility. With this method, monomer solubilities of 2 mM and 7 μM in water were determined for A_8_K and A_10_K, respectively.^13^ The scattered intensity data for MeOH and DMF are presented in Figure 7. For A_8_K, the monomer solubilities of about 10 μM were obtained, which means approximately 2 orders of magnitude smaller compared to that in water. Interestingly, approximately the same solubilities were obtained for A_10_K in these solvents. Hence, for this peptide, we found approximately the same *c*_s_ in water, MeOH, and DMF.

In water, the *c*_s_ of A_10_K is almost 2 orders of magnitude smaller than the *c*_s_ of A_8_K. This can be attributed to the hydrophobic effect.^13^ In MeOH and DMF, the hydrophobic interaction is significantly reduced. Here, instead we need to consider the capability to solvate ions, that is, the charges of the monomers and the TFA counterions, and the possibility of the solvents to hydrogen bond to the peptide amide groups.

The structural similarity among the A_8_K and A_10_K aggregates formed in water, MeOH, and DMF would suggest a similar energetic state of the peptide molecules within the fibrils, almost independent on the surrounding solvent. Therefore, the increased relative stability of the aggregates in the nonaqueous solvents suggested by the lower solubility of A_8_K and enhanced fibril growth should be due to a destabilization of the monomer state in solution when changing the solvent from water to MeOH or DMF.

The driving force for the formation of the fibrillar aggregates of these amphiphilic peptides could be thought in terms of three different energetic contributions: hydrophobic interactions, electrostatic interactions and ion solvation, and hydrogen bonds.^7,48^ In water, the aggregation of A_8_K and A_10_K is mainly driven by the hydrophobic interaction between the alanine amino acids, as reflected in the significant lower solubility of A_10_K, which has two hydrophobic alanine residues more than A_8_K. In MeOH and DMF, on the other hand, solubilities are similar. This indicates that it is rather the hydrogen bonding capacity and electrostatics that determine the monomer solubility, properties that are similar for the two peptides.

We can schematically consider how the change of solvent properties, presented in Table 1, when moving from water to slightly less polar MeOH and DMF, can affect the different energetic contributions to fibril formation.

The electrostatic interaction between ions is expected to increase in magnitude when changing the solvent from water to MeOH and DMF because of the lowering of the dielectric constant. This effect should increase the repulsion between the charged peptide molecules and thereby hinder the aggregation. This is not observed in our case where aggregates form and grow even longer in MeOH than in water. It is reasonable that a lower solvating ability of MeOH and DMF toward the charged groups of the peptides and the TFA counterions can lower the ionization degree of the peptides in these solvents, thereby decreasing the peptide–peptide repulsion and favoring the aggregation.

Less polar solvents are expected to decrease the surface energy of the apolar alanine side chains reducing the solvophobic contribution to the self-assembly, which mainly affects the lateral lamination. This phenomenon could explain the slightly smaller optimum number of laminated β-sheets deduced from the fibril cross section in the less polar solvents MeOH and DMF.

Solvents such as MeOH or DMF are less prone than water to hydrogen bonding with the peptide monomer. In these nonaqueous solvents, the interpeptide hydrogen bond formation within the fibril provides a notable energy gain and becomes a key driving force to the peptide assembly into ribbons, especially in the longitudinal direction.

The less favorable hydrogen bonding between the peptide and solvent molecules in MeOH and DMF compared to water could also have an impact on the kinetics of fibril growth. The slow kinetics observed for reaching a steady state for the aggregation of A_10_K fibrils in water would be in agreement with their crystalline nature, which implies a formation mechanism with nucleation and growth. These are both activated processes, for which the energy barrier also involves the replacement of hydrogen bonds for a successful docking of the peptide within the β-sheets.^57^ In this view, the growth of peptide fibrils in length, with elongation of the β-sheets, should also proceed faster in MeOH and DMF. The observation of “enhanced growth” of the peptide fibrils in these solvents probably includes both thermodynamics and kinetics aspects.

### Fibril–Fibril Interactions

**Figure 8:**
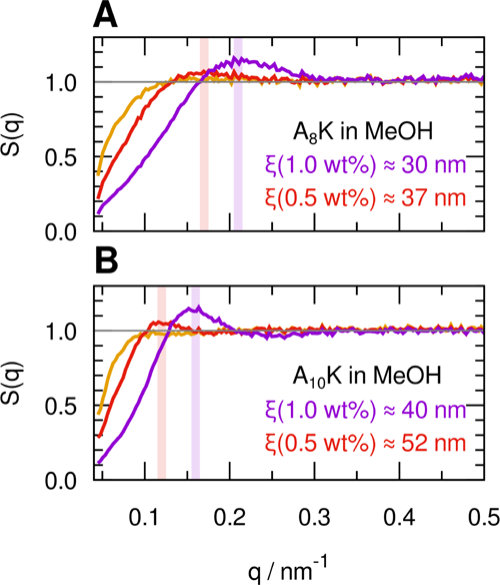
Experimental structure factors of the A_8_K (A) and A_10_K (B) samples in MeOH at concentrations 1 wt % (purple), 0.5 wt % (red), and 0.25 wt % (orange), estimated as the ratio between the SAXS profiles of the samples at higher concentration and that of the sample at 0.12 wt %, which could be identified with the form factor of the aggregates. Correlation lengths estimated from the *q*_peak_ positions as ξ = 2π/*q*_peak_ are reported.

The peak observed in the low *q* range in the SAXS profiles for the peptides in MeOH and DMF at higher concentrations, together with the slow relaxation in DLS experiments, suggested that interparticle interactions are not negligible in this concentration regime. This is in contrast to aqueous samples, where similar peptide mass concentrations could still be considered dilute in the SAXS data interpretation.^13,17,58^ Interparticle interactions can be quantified in terms of an effective structure factor, *S*_eff_(*q*), that can be obtained from scattering data, by dividing the experimental scattering curve with the experimental form factor measured at high dilution, where *S*_eff_(*q*) ≈ 1.^59^ In particular, for a binary system, *S*_eff_(0) reports on the osmotic compressibility
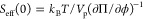
4where Π is the osmotic pressure and *V*_p_ is the particle volume. In Figure 8, we have plotted *S*_eff_(*q*) for A_8_K and A_10_K in MeOH for three concentrations, 0.25, 0.5, and 1.0 wt %. As can be seen, *S*_eff_(0) decreases with increasing concentration, demonstrating that interaggregate interactions are predominantly repulsive. Interestingly, *S*_eff_(0) is indeed significantly lower in MeOH compared to water (see Figure 3A,C). The reason for this is not fully clear.

We expect a stronger counterion condensation in MeOH because of the lower dielectric constant and hence a weaker electrostatic repulsion. However, changes of the potential at the surface of the charged fibrils, involving the protonation state of the ionizable groups, counterions, and solvation effects, are difficult to predict. For A_8_K in water, the pH decrease as a function of increasing concentration has been observed, compatible with a partial deprotonation of the peptides being involved in the formation of aggregates.^60^ This aspect might change when the peptides self-assemble in a nonaqueous solvent. In any case, a stronger steric-excluded volume repulsion in MeOH is expected because of the longer aggregates and their increased overlap.^37^

The peak in *S*_eff_(*q*) reports essentially on the mesh size of the network
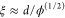
5where *d* is the effective aggregate diameter (≈5 nm) and ϕ is the volume fraction. Experimental estimates of ξ can be obtained from
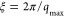
6where *q*_max_ is the position of the structure factor peak. The estimated values (Figure 8) agree reasonably well with the simple formula for ξ, and as expected, ξ decreases with increasing concentration.

## Conclusions

We have shown that the model peptides A_8_K and A_10_K self-assemble into similar ribbonlike aggregates in both MeOH and DMF as in water. However, in the case of A_8_K, the self-assembly begins at roughly 100 times lower concentrations in MeOH and DMF compared to water. We attribute this decrease to a lower degree of hydrogen bonding in the nonaqueous solvents, so that the formation of interpeptide hydrogen bonds in fibril formation becomes a relevant driving force to aggregation.

As in water, DLS experiments indicate a dramatic slowing down of dynamics in MeOH and DMF as the ribbonlike aggregates overlap and form a glassy state. This also occurs at lower mass concentration, compatibly with the idea that fibrils formed in the nonaqueous solvents grow longer. Finally, repulsive interparticle interactions are stronger in the nonaqueous solvents than in water. The reason for this is not yet fully understood but might be sought in the charge state and degree of counterion binding at the aggregate surface, determining different electrostatic potentials, and to the complex interplay between the electrostatic interactions and excluded volume effects for highly anisotropic particles.

## Supporting Information Available

SAXS data of dilution series, sample concentrations, results of SAXS data fitting and model-independent analysis, WAXS data in DMF, and estimate of aggregate thickness from Cryo-EM micrographs (PDF)
