# Supplementary material for: Self-Assembly of Model Amphiphilic Peptides in Nonaqueous Solvents: Changing the Driving Force for Aggregation Does Not Change the Fibril Structure
Source: Langmuir. 2020 Jun 28;36(29):8451–60. doi: 10.1021/acs.langmuir.0c00876 (PMC8009514; doi:10.1021/acs.langmuir.0c00876)
Supplement: Supplementary file 1 — la0c00876_si_001.pdf [file la0c00876_si_001.pdf]

Supporting information

Self-assembly of model amphiphilic peptides in non-aqueous solvents: changing driving forces, same structure?

Alessandra Del Giudice,*,† Axel Rüter,*,‡, Nicolae Viorel Pavel,† Luciano Galantini,† and Ulf Olsson‡

† *Department of Chemistry, Sapienza University of Rome, P. le A. Moro 5, 00185 Rome, Italy.*

‡ *Division of Physical Chemistry, Lund University, SE-22100 Lund, Sweden.*

Email: alessandra.delguidice@uniroma1.it; axel.ruter@fkem1.lu.se

Number of pages: 5

Number of figures: 4

Number of schemes: 0

Number of tables: 3

List of Tables

S1 Concentration of samples of the dilution series S2

S2 SAXS model fitting results S3

S3 Estimates about the cross section of the fibrils S3

List of Figures

S1 SAXS data of dilution series in MeOH on absolute scale S4

S2 PDDF of the cross section from SAXS S4

S3 WAXS data S5

S4 Thickness estimate from CryoEM S5

Table S1 The scaling constants used for plotting the data in the insets of Figure 3A,C are tabulated (last column) together with the experimental concentrations in wt%, the estimated volume fractions (ϕ), and the ratios of the concentration between the 1 wt% sample chosen as a reference and the more dilute samples (1/Ratio wt%)

Sample	wt%	ϕ*	1/ Ratio wt%	Scaling constant**
A_8K_MeOH-Lab	1.01	0.0053	1	1.2
A_8K_MeOH	1.01	0.0053	1	1
	0.49	0.0026	2.1	2.3
	0.26	0.0014	3.9	4.8
	0.12	0.0006	8.2	10.6
	0.06	0.0003	16.7	17.9
A_8K_DMF-Lab	1	0.0063	1	1.3
A_8K_DMF	1.03	0.0065	1	1
	0.5	0.0032	2.1	2.2
	0.25	0.0016	4.2	5.6
	0.12	0.0008	8.4	11.2
	0.06	0.0004	16.7	24.2
$A_{10}K$_MeOH-Lab	1.03	0.0065	1	1.2
$A_{10}K$_MeOH	1.05	0.0066	1	1
	0.52	0.0033	2	2.3
	0.25	0.0016	4.2	4.8
	0.12	0.0008	8.7	9.8
	0.06	0.0004	16.5	17.9
$A_{10}K$_DMF-Lab	0.99	0.0075	1.1	1.3
$A_{10}K$_DMF	1.04	0.0079	1	1
	0.52	0.0039	2	2.3
	0.26	0.0019	4.1	4.7
	0.13	0.001	8.2	15.2
	0.06	0.0005	17.3	33.9

*estimated considering a peptide density in all solvents of 1.26 g (without counterions)/ml for $A_{10}K$ and 1.50 g/ml for A_8K.
**calculated to optimize superimposition in the q range 0.635-1.227 nm^{-1}

Table S2 Results of the fitting of the A_8K and A_{10}K SAXS data shown in Figure 3. The form factor of an elliptical cylinder was used (http://www.sasview.org/docs/user/models/elliptical_cylinder.html). The scattering length densities were fixed: $11.4{\cdot}10^{-4}$ nm^{-2} for the cylinder, $7.5{\cdot}10^{-4}$ nm^{-2} for MeOH, $8.8{\cdot}10^{-4}$ nm^{-2} for DMF and $9.4{\cdot}10^{-4}$ nm^{-2} for water.

Sample	Fixed	q Range (nm^{-1})		Optimized				χ^2
	L (nm)	q_{min}	q_{max}	scale	bg	radius (nm)	axis ratio	
A_8K H_2O 1.35 wt%	350	0.1	3.5	$8 \cdot 10^{-3}$	$2 \cdot 10^{-3}$	1.64	2.3	7.2
A_8K MeOH 0.12 wt%	350	0.1	3.5	$9 \cdot 10^{-4}$	$9 \cdot 10^{-4}$	1.33	2.2	1.5
A_8K DMF 0.25 wt%	350	0.06	3.5	$2 \cdot 10^{-3}$	$7 \cdot 10^{-5}$	1.33	2.4	1.8
A_{10}K H_2O 1.00 wt%	60	0.1	3.5	$1 \cdot 10^{-2}$	$5 \cdot 10^{-4}$	1.75	3.2	5.5
A_{10}K MeOH 0.12 wt%	350	0.1	3.5	$8 \cdot 10^{-4}$	$5 \cdot 10^{-4}$	1.57	2.5	9.3
A_{10}K DMF 0.12 wt%	350	0.1	3.5	$7 \cdot 10^{-4}$	$3 \cdot 10^{-5}$	1.57	2.9	4.4

Table S3 Values of the radius of gyration of the cross-section R_{CS} of a long rod, obtained from the SAXS data of A_8K and A_{10}K by applying a Guinier fit for rod-like objects to the scattering intensity multiplied by the scattering vector $I(q) \cdot q$ in the q range indicated. From this, the number of laminated β sheets constituting the fibril cross section (N) is estimated within the approximation of an elliptical cross section with semi axes a and b, for which $R_{CS} = \sqrt{(a^2 + b^2)/2}$, and considering $2a = 3.2$ nm for A_8K and 3.9 nm for A_{10}K, and $2b$ as $0.54 \cdot N$ nm.

Sample	q_{min} (nm^{-1})	q_{max} (nm^{-1})	R_{CS} (nm)	$2b$ (nm)	N	N/N_{water}
A_8K H_2O 1.30 wt%	0.24	0.51	2.0	7.4	14	
A_8K MeOH 0.12 wt%	0.25	0.63	1.5	5.3	10	0.72
A_8K DMF 0.25 wt%	0.28	0.59	1.7	6.0	11	0.81
A_{10}K H_2O 1.00 wt%	0.30	0.50	2.5	9.4	17	
A_{10}K MeOH 0.12 wt%	0.33	0.50	2.0	7.1	13	0.76
A_{10}K DMF 0.12 wt%	0.31	0.49	2.3	8.3	15	0.89

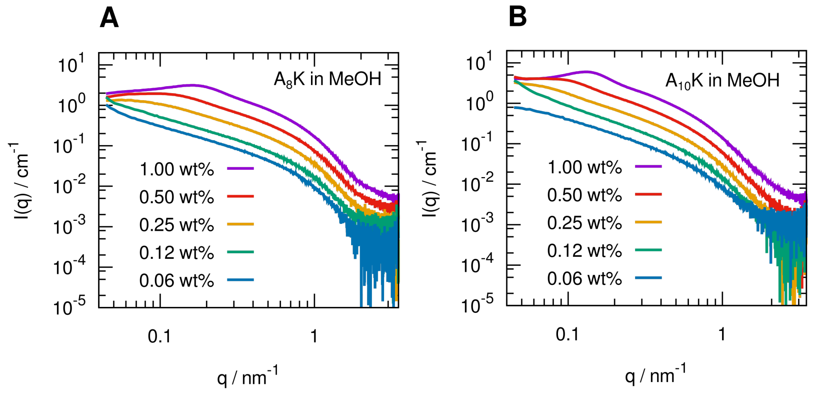

Figure S1 SAXS profiles of A_8K (A) and $A_{10}K$ (B) solutions in MeOH at different concentrations obtained by dilution (approximate concentrations are 1 wt% purple, 0.5 wt% red, 0.25 wt% orange, 0.125 wt% green, 0.0625 wt% blue)

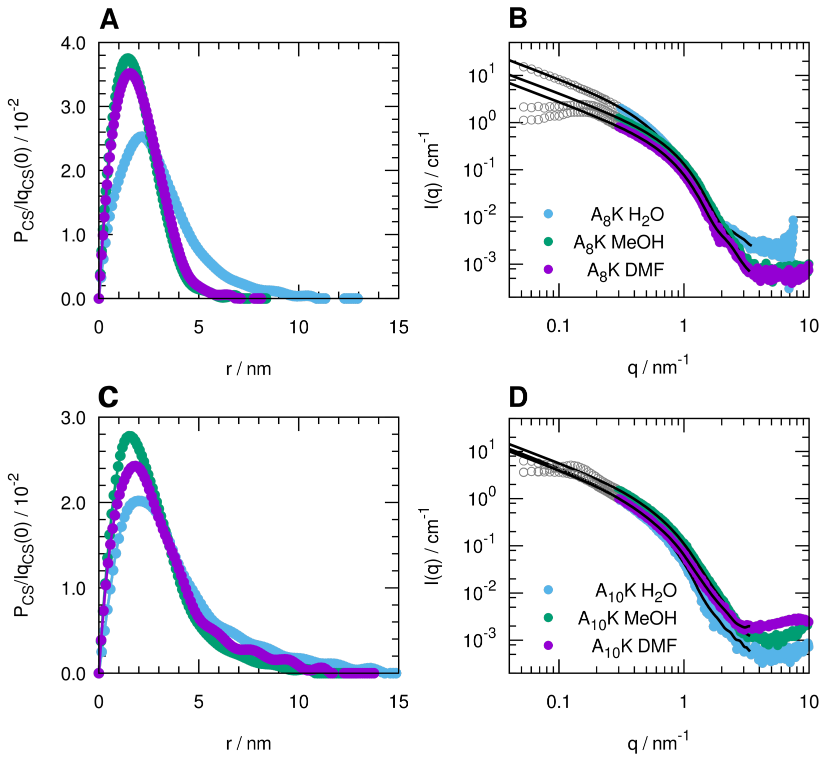

Figure S2 Pair distance distribution functions of the cross section of a long rod obtained by applying the indirect Fourier inversion to the $I(q)q$ experimental profiles of A_8K (A) and $A_{10}K$ (C) fibrils in the solvents water (blue), MeOH (green) and DMF (purple). The distributions are normalized by the subtended area. Fits to the experimental data are shown in (B) (A_8K) and (D) ($A_{10}K$). Sample concentrations are 1 wt% for $A_{10}K$ in all solvents and for A_8K in MeOH and DMF; the data for A_8K in water correspond to a sample with 3 wt% concentration.

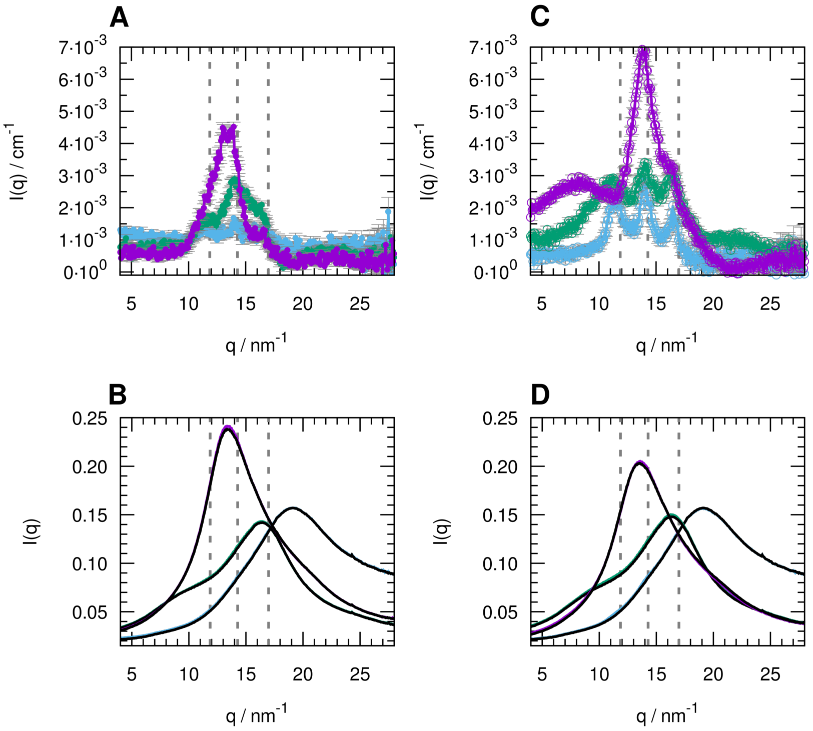

Figure S3 WAXS data of A_8K (A,B) and $A_{10}K$ (C,D) in water (blue), MeOH (green) and DMF (purple) at concentrations around 1 wt%. The q values where Bragg peaks were previously observed are marked with vertical dashed lines. In (B) and (D) the raw data before background subtraction are shown to highlight the solvent signal, which is reported as black lines (almost overlapping).

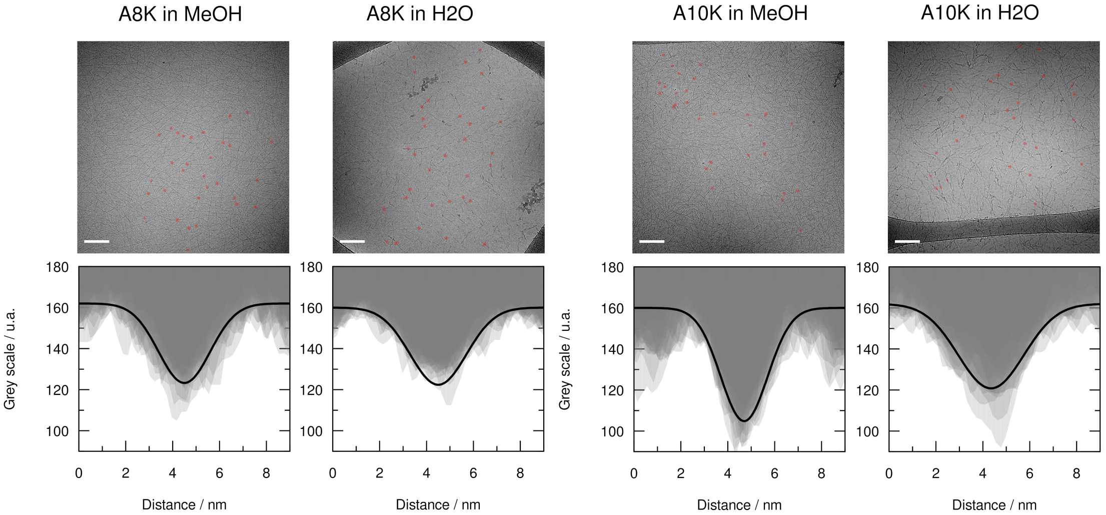

Figure S4 Estimate of the aggregate thickness from the superimposition of several profiles taken across the cross section in several points of the micrographs (red shadows).
